# Orthodeoxia without Platypnea in Hereditary Hemorrhagic Telangiectasia in the Presence of a Cerebral Abscess and Multiple Pulmonary Arteriovenous Malformations: Unusual Complications and Transcatheter Endovascular Treatment

**DOI:** 10.1155/2017/8274981

**Published:** 2017-10-12

**Authors:** Carlos Salazar, Jacky Bruce Blank, Veronica Palmero

**Affiliations:** ^1^Weiss Memorial Hospital, Chicago, IL, USA; ^2^Department of Pulmonary Medicine, South Miami Hospital, Baptist Health, South Miami, FL, USA

## Abstract

Hereditary hemorrhagic telangiectasia is a rare autosomal-dominant condition affecting visceral blood vessel development. Cerebral and most commonly pulmonary arteriovenous malformations are found in the majority of symptomatic patients. The most common complications include embolic strokes and cerebral abscesses, which have been attributed to abnormal vessel communications. Platypnea orthodeoxia is a rare condition that presents dyspnea and oxygen desaturation when adopting an upright position and is relieved on decubitus. The association between hereditary hemorrhagic telangiectasia, pulmonary arteriovenous malformations, and platypnea orthodeoxia has been described in medical literature; however, orthodeoxia as a single entity without platypnea has not been described yet, especially associated with complications of this hereditary condition. We present the case of a 38-year-old male with persistent headaches, in whom a cerebral lesion was detected. Orthostatic tachycardia and severe orthodeoxia without platypnea were evidenced during physical examination. The patient was subsequently diagnosed with hereditary hemorrhagic telangiectasia and underwent cerebral abscess drainage as well as transcatheter endovascular closure of multiple pulmonary arteriovenous malformations. For this reason, the concept of platypnea orthodeoxia syndrome needs further revision. Patients presenting refractory hypoxemia should warn physicians to initially evaluate their oxygen saturation measurements during standing and decubitus position, even though platypnea may not be present.

## 1. Introduction

Hereditary hemorrhagic telangiectasia (HHT) is a rare autosomal-dominant hereditary disease presenting as a vascular dysplasia of multiple organs [1]. The prevalence rate in all races suggested in some population studies is 1 in 10,000, and 2 mutations have been identified, occurring in 85% of cases: type 1 is related to a mutation in the endoglin gene (ENG, chromosome 9q34.1), and type 2 is related to a mutation in the activin receptor-like kinase 1 gene (ACVRL 1, ALK1, chromosome 12q31.34), with a mild phenotype and late onset [2]. Potential neurologic complications of HHT are attributed to pulmonary arteriovenous malformations (PAVMs) and include embolic stroke and cerebral abscess [3]. Platypnea orthodeoxia, defined as dyspnea and hypoxemia when adopting an upright position and resolving during decubitus, has been attributed to PAVMs [4]. We present the case of a male patient diagnosed with HHT, acutely complicated with a cerebral abscess, orthostatic tachycardia, and severe orthodeoxia without platypnea. Positive outcomes were obtained after PAVMs closure.

## 2. Case Report

**Figure 1:**
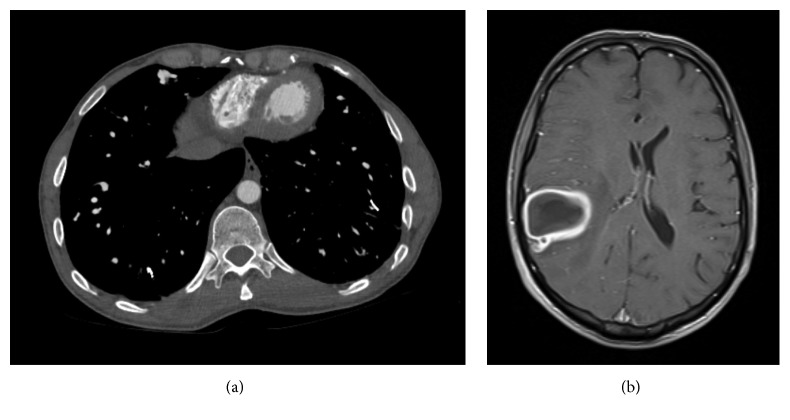
Computed tomography of chest showing multiple PAVMs in right and left lobes (a). T1 weighted postcontrast cerebral MRI showing 4.7 × 3.9 × 5.5 cm ring enhancing lesion located in the right parietal lobe with surrounding edema (b).

A 38-year-old male with a previous history of pulmonary coil embolisation during adolescence was transferred to our facility with a one-week history of persistent headaches and a multiple family history of HHT, manifested as mucocutaneous telangiectasia, bleeding disorders, and cerebral arteriovenous malformations. Vital signs showed orthostatic tachycardia and severe orthodeoxia; however, platypnea was not described (Table 1). A physical examination revealed finger clubbing, peripheral cyanosis, and cutaneous telangiectasias. The rest of the examination was unremarkable. An arterial blood gas (ABG) analysis reported pH: 7.42, pCO_2_: 26.1, and PO_2_: 45.1, and laboratory data exhibited polycythemia (Table 2). Computed tomography of the chest evidenced hyperexpanded lung fields with increased interstitial markings and diaphragmatic flattening. Coil embolisation devices were observed in both the lower lobes. Additionally, areas of mild upper lobes paraseptal emphysema and multiple dilated vascular channels in both lower lobes were prominent. A magnetic resonance image of the brain showed a cerebral lesion, highly suspicious of cerebral abscess (Figure 1).

**Figure 2:**
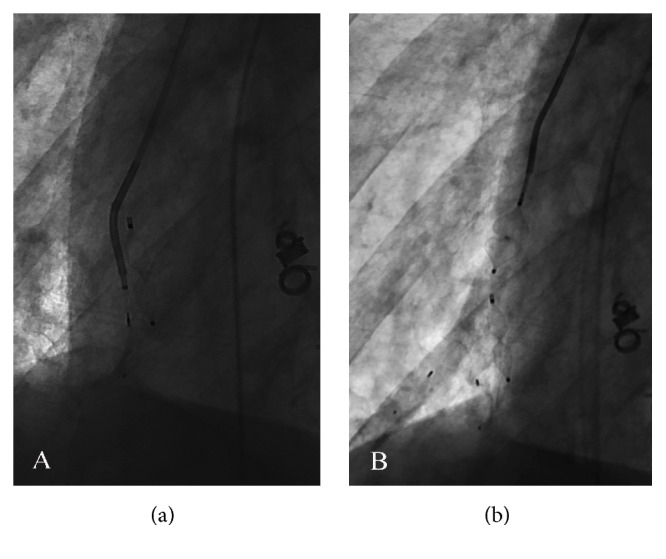
Fluoroscopic image showing deployment of Micro Vascular Plugs through a microcatheter adjacent to an Amplatzer Vascular Plug 4 (a). Fluoroscopic image showing deployment of Amplatzer Vascular Plug 4 through a 4-F 0.037 in catheter (b).

**Figure 3:**
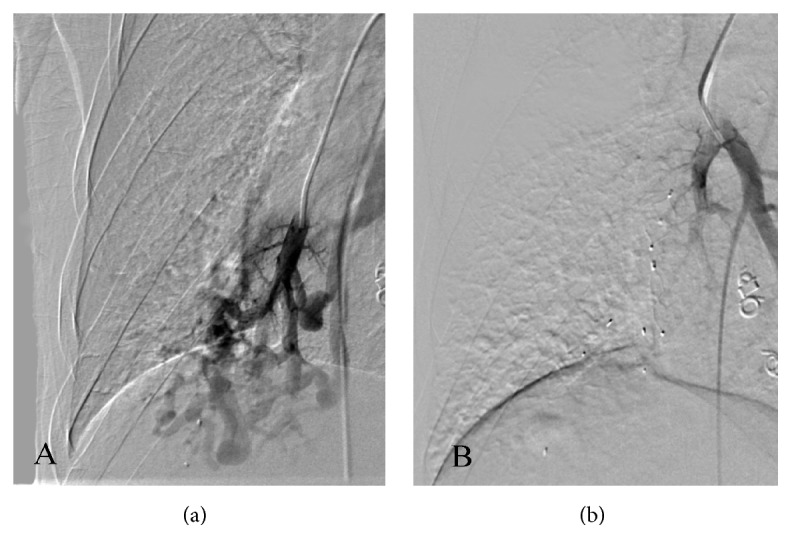
Pulmonary angiogram showing multiple right PAVMs before endovascular closure (a). Pulmonary angiogram showing successful endovascular closure of multiple right PAVMs with subsequent decrease in shunting (b).

The patient was clinically diagnosed with HHT and a triple antibiotic therapy was initiated. Right parietal craniotomy for excision and drainage of cerebral abscess was performed. Following abscess management, a transthoracic echocardiogram revealed a probable right-to-left shunt on agitated saline test, suggestive of pulmonary origin. PAVMs were closed using Amplatzer Vascular Plugs 4 and Micro Vascular Plug System (Figure 2). A significant decrease in orthodeoxia was achieved (Table 1). No peripheral cyanosis was noted, and imaging tests after PAVMs closure revealed considerable improvement (Figure 3). The patient was discharged with follow-up tests and was encouraged to contact family members for screening.

## 3. Discussion

While genetic tests are presently available, the diagnosis of HHT remains clinical and is based on the Curacao criteria which includes epistaxis as well as telangiectasia in the lips, oral cavity, fingers, or nose; visceral, pulmonary, hepatic, cerebral, or spinal AVMs; and affected first degree relative [5]. In patients with HHT, cerebral abscesses arise in the presence of uncontrolled right-to-left shunt, facilitating septic thrombus embolisation through PAVMs. Bacteria could easily break through the cerebral or pulmonary capillary blood barrier, resulting in recurrent and severe cerebral abscesses [6]. Platypnea orthodeoxia has been related to PAVMs due to a right-to-left shunt that strengthens desaturation in the upright position [7]. Orthodeoxia, in this setting, may be related to gravity's effect on the redistribution of pulmonary blood flow to PAVMs located in lung bases (53–70%) [8]. Santhirapala, in a prospective 8-year study, evaluated 257 patients with PAVMs attributed to HHT, and 29% demonstrated orthodeoxia; nevertheless, none of them described platypnea [9]. Some hypotheses suggest that compensatory mechanisms such as polycythemia and postural orthostatic tachycardia may be able to maintain sufficient tissue oxygen delivery during orthodeoxia, explaining the absence of acute platypnea [10, 11]. These literature findings correlate completely with the orthodeoxia and compensatory mechanisms observed in our patient. Pulmonary angiography is considered the gold standard diagnosis for screening and precise localisation of PAVMs [12]. Vascular plugs are effective devices for occlusion of high-flow abnormal vessels in which traditional devices could be technically challenging to use [13, 14]. Ethanol embolotherapy as well has proved to be curative by a combination of a direct denuding effect on the vascular wall and clumping of damaged erythrocytes and denatured proteins, which result in complete obliteration of the vessel lumen preventing recurrences [15].

## 4. Conclusion

PAVMs underline the necessity of immediate closure to prevent cerebral septic embolism and significantly decrease orthodeoxia. Compensatory pathophysiologic mechanisms such as polycythemia and orthostatic tachycardia in patients diagnosed with HHT and PAVMs could decrease the manifestation of platypnea, separating orthodeoxia into a single entity. For this reason, the concept of platypnea orthodeoxia syndrome needs further revision. Patients presenting with refractory hypoxemia should warn physicians to initially evaluate their oxygen saturation measurements during standing and decubitus position, even though platypnea may not be present.

## Figures and Tables

**Table 1:** Oxygen saturation and heart rate.

Positional changes	Admission		Post PAVMs closure	
	Sat O_2_	Heart rate	Sat O_2_	Heart rate
	(%)	(bpm)	(%)	(bpm)
Decubitus	90	89	92	78
Standing	68	124	89	86

Sat O_2_: saturation of oxygen; bpm: beats per minute.

**Table 2:** Complete blood count.

	Admission	Normal values
Hgb (g/dL)	19.1	13–17
Hct (%)	57.4	38–50
RBC (M/uL)	5.97	4–5.7
WBC (K/uL)	7.10	3.4–11
Platelets (K/uL)	203	130–360
MCV (fL)	93	80–100
MCH (pg)	34	26–35
MCHC (g/dL)	36	32–36
RDW (%)	12.8	11–14
MPV (fL)	9.6	7–13
Neutrophils (K/uL)	6.1	1–8
Lymphocytes (K/uL)	0.8	0.6–3.1

Hgb: hemoglobin; Hct: hematocrit; RBC: red blood cell; WBC: white blood cell; MCV: mean corpuscular volume; MCH: mean corpuscular hemoglobin; MCHC: mean corpuscular hemoglobin concentration; RDW: red cell distribution width; MPV: mean platelet volume.

## Abbreviations

HHT: Hereditary hemorrhagic telangiectasia
PAVMs: Pulmonary arteriovenous malformations.

## References

[1] Kjeldsen A. D., Vase P., Green A. (1999). Hereditary haemorrhagic telangiectasia: a population-based study of prevalence and mortality in Danish patients. *Journal of Internal Medicine*.

[2] Boza J. C., Dorn T. V., de Oliveira F. B., Bakos R. M. (2014). Case for diagnosis. *Anais Brasileiros de Dermatologia*.

[3] Donaldson J. W., McKeever T. M., Hall I. P., Hubbard R. B., Fogarty A. W. (2015). Complications and mortality in hereditary hemorrhagic telangiectasia. *Neurology*.

[4] Akin E., Krüger U., Braun P. (2014). The platypnea-orthodeoxia syndrome. *European Review for Medical and Pharmacological Sciences*.

[5] Shovlin C. L., Guttmacher A. E., Buscarini E. (2000). Diagnostic criteria for hereditary hemorrhagic telangiectasia (Rendu-Osler-Weber syndrome). *American Journal of Medical Genetics*.

[6] Dong S. L., Reynolds S. F., Steiner I. P. (2001). Brain abscess in patients with hereditary hemorrhagic telangiectasia: case report and literature review. *The Journal of Emergency Medicine*.

[7] Kumar N., Kraemer R. R., Murthy R. K., Hartig J. R. (2012). Platypnea-orthodeoxia syndrome as a presentation of hereditary hemorrhagic telangiectasia. *Circulation*.

[8] Bosher L. H., Blake D. A., Byrd B. R. (1959). An analysis of the pathologic anatomy of pulmonary arteriovenous aneurysms with particular reference to the applicability of local excision. *Surgery*.

[9] Santhirapala V. (2014). Orthodeoxia and postural orthostatic tachycardia in patients with pulmonary arteriovenous malformations: a prospective 8-year series. *Thorax*.

[10] Santhirapala V., Williams L. C., Tighe H. C., Jackson J. E., Shovlin C. L. (2014). Arterial oxygen content is precisely maintained by graded erythrocytotic responses in settings of high/normal serum iron levels, and predicts exercise capacity: An observational study of hypoxaemic patients with pulmonary arteriovenous malformations. *PLoS ONE*.

[11] Cottin V., Chinet T., Lavolé A. (2007). Pulmonary arteriovenous malformations in hereditary hemorrhagic telangiectasia. *Medicine*.

[12] Barzilai B., Waggoner A. D., Spessert C., Picus D., Goodenberger D. (1991). Two-dimensional contrast echocardiography in the detection and follow-up of congenital pulmonary arteriovenous malformations. *American Journal of Cardiology*.

[13] Wiegand G., Sieverding L., Bocksch W., Hofbeck M. (2013). Transcatheter closure of abnormal vessels and arteriovenous fistulas with the amplatzer vascular plug 4 in patients with congenital heart disease. *Pediatric Cardiology*.

[14] Saluja S., Sitko I., Lee D. W., Pollak J., White R. I. (1999). Embolotherapy of pulmonary arteriovenous malformations with detachable balloons: long-term durability and efficacy. *Journal of Vascular and Interventional Radiology*.

[15] Young S. D., Yakes W. F., Sung W. S. (2005). Ethanol embolization of arteriovenous malformations: interim results. *Radiology*.

